# Nanodrug Delivery Systems for the Treatment of Ovarian Cancer

**DOI:** 10.3390/cancers12010213

**Published:** 2020-01-15

**Authors:** Jonathan M. Pantshwa, Pierre P. D. Kondiah, Yahya E. Choonara, Thashree Marimuthu, Viness Pillay

**Affiliations:** Wits Advanced Drug Delivery Platform Research Unit, Department of Pharmacy and Pharmacology, School of Therapeutic Sciences, Faculty of Health Sciences, University of the Witwatersrand, Johannesburg, 7 York Road, Parktown 2193, South Africa; monwabisi.pantshwa@wits.ac.za (J.M.P.); pierre.kondiah@wits.ac.za (P.P.D.K.); yahya.choonara@wits.ac.za (Y.E.C.); thashree.marimuthu@wits.ac.za (T.M.)

**Keywords:** nanosystems, drug delivery, nanomicelles, ovarian cancer, tumour targeting, chemotherapeutics

## Abstract

Despite advances achieved in medicine, chemotherapeutics still has detrimental side effects with ovarian cancer (OC), accounting for numerous deaths among females. The provision of safe, early detection and active treatment of OC remains a challenge, in spite of improvements in new antineoplastic discovery. Nanosystems have shown remarkable progress with impact in diagnosis and chemotherapy of various cancers, due to their ideal size; improved drug encapsulation within its interior core; potential to minimize drug degradation; improve in vivo drug release kinetics; and prolong blood circulation times. However, nanodrug delivery systems have few limitations regarding its accuracy of tumour targeting and the ability to provide sustained drug release. Hence, a cogent and strategic approach has focused on nanosystem functionalization with antibody-based ligands to selectively enhance cellular uptake of antineoplastics. Antibody functionalized nanosystems are (advanced) synthetic candidates, with a broad range of efficiency in specific tumour targeting, whilst leaving normal cells unaffected. This article comprehensively reviews the present status of nanosystems, with particular emphasis on nanomicelles for molecular diagnosis and treatment of OC. In addition, biomarkers of nanosystems provide important prospects as chemotherapeutic strategies to upsurge the survival rate of patients with OC.

## 1. Introduction

Globally, ovarian cancer (OC) is a lethal condition that accounts for millions of deaths annually in females, making this condition a major health issue [1,2,3,4,5,6]. In the last five-year survey, statistics reflected approximately 21.9 million new patients clinically diagnosed with OC on a yearly basis, with 14,270 deaths predicted in the United States every year [7]. According to the World Health Organization (WHO), OC is one of the most lethal genital malignancies in females in developing countries, with this asymptomatic disease exacerbated by lack of early diagnostic strategies and access to expensive chemotherapeutic drugs [1]. In Africa (South Africa), the Cancer Council of Southern Africa (CANSA) confirmed more than 500 cases of OC [8]. Globally, the five-year survival rate ranges from 15%–20% for the population with advanced stage ovarian cancer, even though patients undergo operative surgery and platinum chemotherapy [9].

The treatment of OC employs invasive surgery for the removal of infected ovaries, uterus, fallopian tubes, cervix and lymph nodules in the abdomen. The surgical approach is followed by external beam radiotherapy or systemic chemotherapy, depending on the stage at which the OC disease is identified. Intravenous paclitaxel and alkylating cisplatin are conventional therapeutics employed for treatment of OC with antimetabolite methotrexate also considered as a possibility. However, conventional treatment has its own share of drawbacks, including toxicities and subsequent disease relapse, due to the development of multidrug resistance. In addition, the chemodrug is not specific for OC destruction, hence exhibiting dose cytotoxicity [10,11,12]. Furthermore, the long-term prognosis is usually adversative with expression and development of chemoresistant tumours. Patients undergo diverse side effects including excessive nausea, hair loss and deterioration in plasma cell counts linked with the administration of chemotherapy for OC treatment [13]. To circumvent treatment drawbacks of conventional antineoplastics, several targeted drug delivery platforms have been developed to direct antineoplastics to specific tumour sites [14].

New advances in polymeric nanotechnology—with particular emphasis on nanomicelles—provide feasible alternatives for early detection and targeted treatment of metastatic OC, thereby minimizing systemic toxicity associated with administration of chemotherapeutic drugs. The nanosystems employed as theranostics include polymeric nanoparticles, nanomicelles, nanoconjugates, as well as dendrimers [14,15,16].

**Figure 1:**
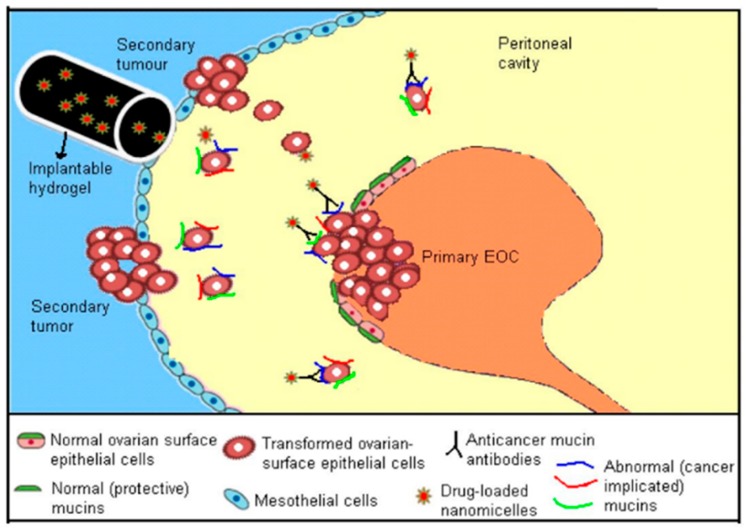
Illustration of the expression of cancer-associated mucins, accompanying the development of ovarian cancer and the intraperitoneal implant treatment, providing targeted therapy within the peritoneal cavity (Adapted with permission from [21].

In order to improve diagnosis and chemotherapeutic efficacy in ovarian cancer treatment, this article presents a critique of, (a) formulation of nanoparticulate delivery systems (including nanomicelles), and (b) nanoparticulate delivery systems functionalized with ligands such as antibodies to expedite specific elimination of tumours, imaging analysis and aid in decreasing drug-related side effects (Figure 1). Thus, the basics of design of these delivery systems are to improve blood circulation in vivo, polymeric biodegradability, and theranostic compatibility with adequate retention time, for nanocarrier-related therapeutics. Furthermore, the synthetic building blocks of the carrier systems are nontoxic, noninteractive with inflammatory responses, and biocompatible. Other significant properties of polymeric carrier systems are biodegradability and clearance by hepatic/ renal pathways post-drug release, with the prospect to be further traced with additional benefits in molecular imaging technologies [17].

This review thus aims to present advances in nanosytems-based molecular diagnosis and treatment of OC. A particular focus is on nanomicelles as one of the most researched nanoarchetypes for imaging/diagnosis and targeted OC treatment. The status of OC biomarkers is concise, with the integration of studies conducted on mucins and possible application in early diagnostics and management of OC. These approaches are defined to potentially identify the disease at an early stage, halt disease progression and promote recovery.

## 2. Current Nano-Based Drug Delivery Approaches for Ovarian Cancer Theranostic

Numerous nanodrug delivery vehicles have been developed including nanoconjugates, branched dendrimers, liposomes, nanostructured lipid formulations and polymer nanomicelles (Table 1) [18,19]. These drug delivery systems have many advantages including the promotion of therapeutic drug delivery and fulfilling several (biopharmaceutical) parameters, such as a marked increase in therapeutic impact compared to the free drug, good biodegradability and biocompatibility, nontoxic and noninflammatory characteristics, as well as future prospects in scaling-up manufacturing [20]. In chemotherapeutic systems, a nanoformulation must possess high drug-loading capacity, the ability to dissolve drugs within the inner core and selectively accumulate in tumour tissue through permeability and retention influence (passive or active targeting). Targeted chemotherapy, such as intraperitoneal implantable treatment, provides targeted therapy within the peritoneal cavity (Figure 1) [21,22,23,24,25]. In addition, the preparation of nanoformulations functionalized with specific ligands facilitates preferential targeting of OC tumours and ultimately increases the therapeutic effect in comparison to nonfunctionalized nanosystems [25,26,27,28,29,30].

In this context, there is a significant need to develop stable molecular biomarkers for early detection of OC [14]. Various prospective biomarkers of OC are reported. Epithelial ovarian tumours display modified cell antigens including, Human Epididymis Protein 4 (*HE4* gene), Cancer Antigen *72-4*(CA 72-4), Renal Estimated Glomerular Filtration Rate (EGFR), Soluble Mesothelin-Related Peptides (SMRP), Mesothelin, Osteopontin (OPN), Alpha-Fetoprotein (AFP), Cytotoxic T-Lymphocyte-associated Protein 4 (CTLA4), Interferon-alpha (IFNα), Kallikrein-6 (KLK6), phospholipase A2 group 2A (PLA2G2A), Erythroblastic Oncogene B2 (ErbB2), Interleukin-10 (IL-10) and Mucin-16 (MUC1–16), that differentiate cancerous cells from healthy ovarian tissue and other ordinary cells covering the intraperitoneum [23,24,25,26,27,28,29,30]. Mucin proteins (specifically MUC16) show prospective as biomarkers and antibody functionalized micelles to provide a broad range of prospects for OC therapy [32].

Nanomicelles are synthetic nanovehicles, with high potential loading capacity for chemotherapeutics designed for site-specific ovarian tumour targeting [33,34,35,36]. A size range of a micelle between 10–100 nm promotes significant permeability, endocytosis by OC cells and decreases nonselective targeting of normal cells [37]. Nanomicelles can perforate and assemble in regions with permeated vasculatures, including tumours and inflamed tissues [37,38,39]. In addition, improved biocompatibility, in vivo stability, ability to incorporate a wide range of hydrophobic chemotherapeutics, as well as extended plasma circulation periods, are achieved [40,41].

## 3. Critical Comparison of Nanosystems to Nanomicelles for OC Treatment

**Figure 2:**
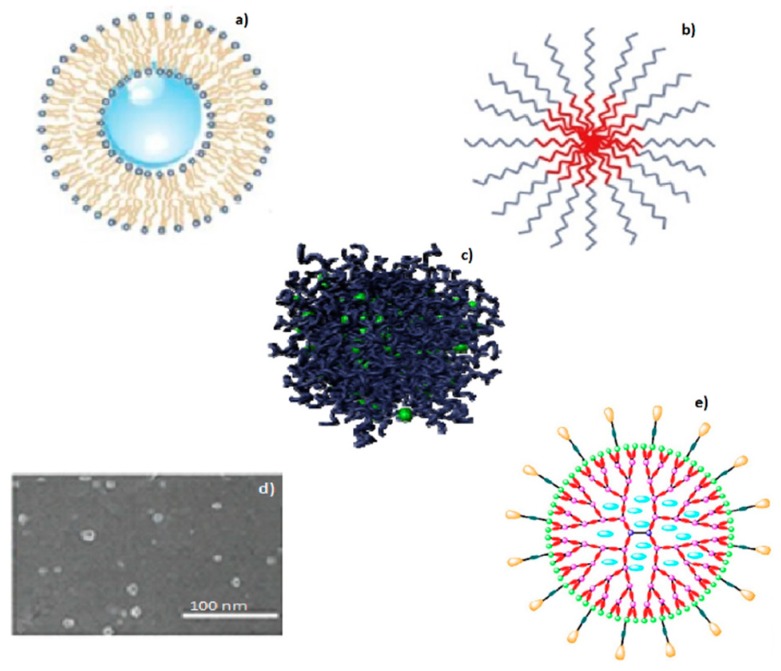
Schematic depicting examples of nanosized delivery systems; (**a**) liposomes, (**b**) nanomicelles, (**c**,**d**) polymer–drug conjugates, and (**e**) dendrimers, which are currently explored in detail for transport of chemotherapeutic agents (adapted with permission from (**a**) Trucillo et al. [31], (**b**) Brandta et al. [51], (**c**,**d**) Tong et al. [52], (**e**) Huang &Wu [53].

Polymer–drug conjugates or prodrugs are macromolecular dispersed systems that require covalent binding of the active principle while nanoparticles on the contrary are physically attached to the active principle. Polymer–drug conjugates have low molecular weight (specific to polymer incorporation), which permits molecular targeting within the cancer cell [42,43]. Physicochemical properties of polymer–drug conjugates (pH, enzymatic-alteration, acid (H^+^)-catalytic chemical reactions) are vital for drug release at a tumour site. Polymer–drug conjugates are extensively evaluated for prolonged drug release in cancer cells, tumour mass invasion, and enhancement of anti-tumour proliferation [44,45,46]. Therapeutics in ovarian cancer also utilize branched dendrimers formulated from several polymers and genetic DNA, however acrylamide branched nanodendrimers are usually utilized [46]. Branched dendrimers have characteristic design components including (i) peripheral surface with several potential attachment sites, (ii) the central inner core where diverse dendrons demarcate the alienated constituent stratums covering the inner core and (iii) the location for dendronic conjugation. The three fragments of branched dendrimers are modified for several uses, including drug transport and DNA delivery [47]. Polymer–drug conjugates and dendrimers have covalently bonded drug molecules to the polymeric carriers. This consecutively necessitates the association of the complex with specific biochemical processes, shielding the complex from in vivo catalytic enzymatic destruction and protonic acid-hydrolytic reactions [48,49,50]. Furthermore, the minute-size of these carriers (normally 10 nm), enables perforation through plasma membranes of the glomeruli [17]. Significant assemblies and differentiating properties of these delivery systems are demonstrated in Table 1, as well as in Figure 2.

Liposomes are similar to nanomicelles due to microscopic spherical shape, consisting of a lipid bilayer, encapsulating aqueous components for hydrophobic drug incorporation. Nonpolar lipophilic drugs are incorporated within the lipid bicoating, while water-soluble drugs reside in the vesicle. Entrapment of antineoplastic therapies in lipophilic liposomes result in pharmacokinetic modifications and pharmacodynamics features, with a resultant decline in drug degradation and improved dose cytotoxicity [31,51,52,53]. These lipophilic nanocarriers can be utilised for specific targeting and imaging of tumour tissues; however, ligands applied to the phospholipid coating improve cellular uptake thus enabling a pronounced therapeutic effect to the targeted specific-sites [31,51,52,53,54]. Solid lipid nanoparticles (SLNs) also have similar properties to nanomicelles. On the contrary, the major challenges with liposomes include instability and difficulty in large-scale synthesis.

Poorly water-soluble drugs are encapsulated in the hydrophobic interior of SLNs, but the distribution ability is hindered by membrane destabilization. However, most liposomal and SLNs are above 90 nm in size, due to intrinsic structural parameters which significantly restrict delivery to ovarian tumour tissue. To surmount the setbacks associated with liposomes and SLNs, other nanoplatforms, including nanoemulsions, polymeric nanoparticles and polymeric nanomicelles are employed [19].

Nanoemulsions are used as templates for polymeric nanoparticle preparation. Therapeutic-loaded nanoemulsions are formed by oil-in-water (o/w) solvent evaporation techniques, employing miscible organic solvents (diethyl ether, chloroform, N, N-dimethylformamide (DMF), acetonitrile, THF). Simple liquid emulsions are either oil-suspended in an aqueous state (o/w), or water-suspended in oil (w/o). Nanoemulsions are aqueous emulsions with sizes normally between 20–200 nm. Nanoemulsions are formulated employing low-energy emulsification procedures, in which the nanosize is adjusted by the physicochemical parameters of the process [55], allowing the development of small-scale and homogeneous droplets, employing a high-energy system, in which a nanosized droplet is adjusted by the degree of the peripheral energy contribution. Among the low-energy emulsification approaches, the Phase Inversion Composition (PIC) system is highly beneficial for structures with thermo-labile composites, including therapeutics, as it can be accomplished at ambient temperature. In the PIC system, the emulsification process is activated by the variations in the voluntary amphiphilic curvature generated in emulsification, changing the constituent at stable temperatures [55].

### Morphology, Composition and Mechanism of the Formation of Nanomicelles

**Figure 3:**
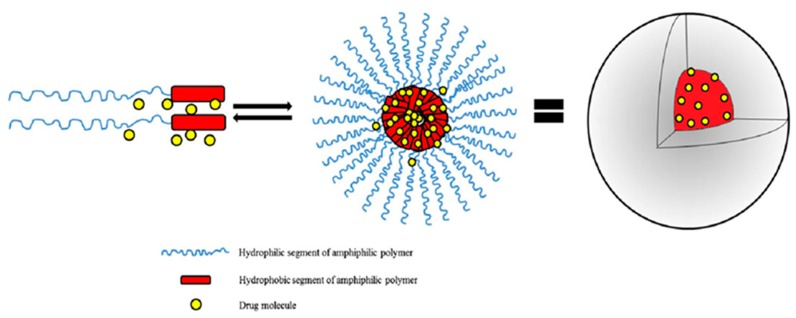
Schematic representation of the supramolecular structure of polymeric micelles (adapted with permission from Lu and Park [47]).

Nanomicelles are spontaneously self-assembled or aggregated as versatile nanoparticles formed in water at certain physicochemical parameters including concentration (above Critical Nanomicelle Concentration-CNC), temperature and conductivity; employing surfactants (hydrophilic–hydrophobic polymers), with opposite affinities towards a particular solution [55]. The assembly of amphiphilic components generates the structure or shape of nanomicelles. The copolymer sequence controls the configuration of the prepared nanomicelles. Thin rod designed nanomicelles are also generated when the water-insoluble component is greater than the water-soluble component. Sphere-shaped nanomicelles are usually indicative of a longer hydrophilic component with a minor hydrophobic component, or possibly a result of equal degrees of the amphiphilic components [56]. Constituents of the prepared supramolecular structure of polymeric micelles are usually di- or tri-segment/block, or a stable copolymer (Figure 3). Poly (ethylene oxide) (PEO) forms a barrier to nanomicelles, collapsing and displaying dissolution in an aqueous solvent [33,57]. The inner central component normally possesses a biodegradable polymer such as PEO/β-amino polyesters that can be utilised as an inner core to dissolve hydrophobic pharmaceutical drugs, thus shielding loaded constituents from the aqueous environment; increasing the bioavailability and in vivo efficacy [58,59,60].

At low ratios in aqueous media, copolymers exist separately, however, once the molar concentration is increased, aggregation occurs [61]. The aggregates known as nanomicelles, comprises of several copolymers in a spherical arrangement [62,63]. When attachments to the polymer functional groups are anticipated, complexes including carboxylic (COO^−^) conjugate bases and amine (NH) bases are joined as the sequence terminating clusters [33].

## 4. Classification of Nanomicelles

Nanomicelles are classified into three key distinct nanosystems i.e., colloidal nanomicelles formulated from an aggregation of polar and nonpolar molecules in an aqueous solution (amphiphilic aggregates), polyionic nanomicelles formulated from oppositely charged polymers generating an agglomeration due to electrostatic interaction, and nanomicelles originating from metal complexation [63,64,65,66].

### 4.1. Amphiphilic Nanomicelles

**Figure 4:**
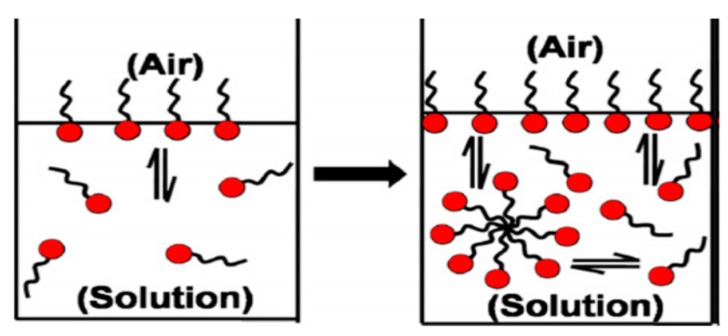
Schematic depiction of surfactant molecules aligning on water/air interface at pre- and post-‘Critical Nanomicelle Concentration (CNC)’ threshold (adapted with permission from Mukherjee et al. [68]).

Amphiphilic colloidal nanomicelles are formed from hydrophobic interactions between the inner core and the outer shell of the surfactant molecules in a solution [60]. An active surfactant retains amphiphilic configurations, comprised of hydrophobic and hydrophilic functional groups [66]. The hydrophilic groups form the polar clusters constructed from several moieties including ionised carboxyl, conjugated sulfonate, ammonium, active hydroxyl and amides. Hydrophobic clusters are nonpolar ends, including molecular hydrocarbon chains with eight or additional carbon molecules, and can be rectilinear or separated structures. Lipophilic and hydrophilic polymers self-assemble into nanomicelles with adequate surfactant concentration. The resultant concentration of surfactant for the formation of nanomicelles is known as critical micelle concentration (CMC). Figure 4 depicts the settings of surfactant alignment on air/aqueous interface to form nanomicelles when subjected to a particular solution with opposite charge affinities to hydrophobic and hydrophilic molecules. The polar ends form the outer surface of nanomicelles with the nonpolar portions, establishes the inner central core. The quantity of drug incorporated into copolymeric nanomicelles is influenced by physicochemical parameters that result in hydrophobic interactions between the drug and the hydrophobic segment of the polymers. Hence, a consideration of physicochemical trends is an invaluable tool in the synthesis of drug loaded copolymeric nanomicelles. The amphiphilic block copolymer, Pluronic^®^ poloxamer, generates amphiphilic nanomicelles in response to electrostatic interactions [63,67].

### 4.2. Polycharged Composite Nanomicelles

Polycharged complex nanomicelles (PCCMs) are formulated by the self-assembly of oppositely charged polymers that form aggregates when distributed in an aqueous solution by hydrophilic groups, usually poly(ethylene glycol) (PEG), and are covalently attached to one of the two ionic polymers. Electrostatic interactions are the intermolecular cohesive forces of the assembled composite; with electrostatic and hydrophobic exchanges employed in formulated nanomicelle complexes. PCCMs are prepared using various synthetic methods, including common synthetic procedures and spontaneous self-assemblage or aggregation in solution. PCCMs are prepared from segment copolymers in an aqueous solution, thus circumventing associated cytotoxicity of organic solvent. These nanomicelles are stable with low CMC values—as low as 10^−6^ M. The central core of PCCMs encapsulates several therapeutics, such as hydrophilic and hydrophobic drugs employing intermolecular cohesive forces and hydrogen bond interactions. Therapeutics such as cisplatin and ionic large-scale drugs are released from PCCMs, subsequent to induction from appropriate stimuli [68].

### 4.3. Noncovalent Connected Polymeric Nanomicelles

These nanomicelles are prepared to employ homopolymeric material or monomer units for nanomicelle agglomeration. The inner and the outer surface are bonded at the polymer edges via specific intermolecular interactions including hydrogen bonds or metal coordinate bonds; for this reason, are known as noncovalently linked nanomicelles. Poly (4-vinylpyridine) functionalized with carboxyl-terminated polybutadiene are the mainstay of intermolecular interaction due to the formation of hydrogen linkages in a common organic solvent such as chloroform [63].

## 5. Surfactants Employed in Nanomicelle Targeted Platforms for Ovarian Cancer

Surfactant nanomicelles utilized for drug delivery have hydrophobic esters, including polypropylene oxide (PPO), poly(L-lactide) (PLA), poly(D,L- lactide) (PDLLA), as well as amino functional groups such as poly lactide-co-gycolide (PLGA), polycaprolactone (PCL), poly (β-amino ester), and polylactic acid (PLA) in their inner core segment for dissolving hydrophobic chemotherapeutics, as illustrated in Table 2. The hydrophobic core segment is compatible, nontoxic, biodegradable and permitted by the U.S. Food and Drug Administration (FDA) for biopharmaceutical application. On the contrary, the soluble hydrophilic corona surface of the nanomicelle used in therapeutic release is composed of poly(ethylene glycol) PEG, poly(ethylene oxide) PEO, poly N-vinyl pyrrolidone (PVP), poly N-isopropyacrylamide (PNIPAM), poly N-vinyl alcohol (PVA), and poly N-2-hydroxyproyl methacrylamide (PHPMAm), as displayed in the first section of Table 2. In this context, the surfactants self-assemble to form micelles in an aqueous solution with the central amino or ester section, which is structurally neutral/uncharged and connected to the hydrophilic corona. Protein copolymers (including drug peptide copolymers) employed in chemotherapeutic delivery enhance the accumulation at pathological sites and improve endocytotic uptake into the tumour cells. Modification of a specific sequence of the amino acid alters enzymatic functioning and the degree of immune system response [53].

## 6. Preparation of Drug-Loaded Nanomicelles for Application in Ovarian Cancer

**Figure 5:**
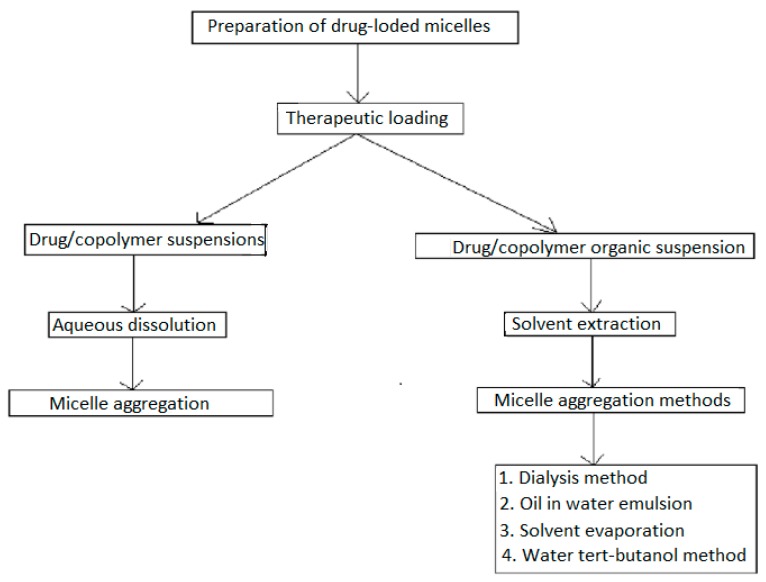
Commonly employed methods of drug-loaded micelle preparation.

Preparation of therapeutic-loaded nanomicelles involves two major categories of therapeutic loading, reliant on the physicochemical properties of a block copolymer (Figure 5) [69]. The first category is the dissolution of co-polymer with a drug in a solution. This method is used in insoluble polymers, including Pluronics poloxamers, and necessitate the warming of the solution for nanomicelle aggregation, utilizing the dehydrated core profiling portion. This dissolution technique is also employed in preparation of PCCMs, with therapeutic polymer dissolved separately, and nanomicelle aggregates impelled by mixing of the two solutions to stabilize therapeutic–polymer ionic proportions [70,71]. The drawback of this technique is the low drug loading that occurs in nanomicelles [60,72].

The second method of therapeutic loading involves the surfactant, which are partially water-soluble and for which an organic solvent (such as, tert-butyl alcohol, ethyl acetic acid, methyl alcohol, toluene, dichloromethane (DCM), aprotic diethyl ether and chloroform/trichloromethane) is required to dissolve the polymer and therapeutic [73]. Nanomicelle aggregation is reliant on the liquid extraction technique. For homogenous solutions, nanomicelle formulations are extracted via a dialysis exchange method, with slow extraction of the organic solvent that activates nanomicelle aggregation. A drawback of the dialysis technique is that the dissolving of a drug–polymer involves the use of chlorinated solvents, which are toxic and thus necessitate extra time (<36 h) for the adequate encapsulation of therapeutics into the nanomicelles. Alternatively, the solvent-evaporation technique is utilized for the removal of organic solvents by air diffusion to form a polymeric film. The introduction of water to the film with heating facilitates the aggregation of drug-loaded nanomicelles. Nanomicelles synthesized from solvent-evaporation technique have increased potential of dissolving high quantities of partially soluble drugs. These methods all require sterilization and freeze-drying stabilization processes for preservation of the synthesized formulations. Figure 5 depicts the drug loading techniques for nanomicelle formulation.

The limitations in preparations of therapeutic-loaded nanomicelles are surmounted by employing improved approaches such as the tert-butanol (TBA) method, which incorporates the solution of copolymer and therapeutic liquid/TBA medium followed by freeze-drying, to form a dry powdered lyophilized cake. Stable nanomicelles spontaneously self-aggregate, upon resuspension of the lyophilized powdered polymer–therapeutic cake in an aqueous solution [74,75].

## 7. Applications of Nanomicelles in Ovarian Cancer

Nanomicelles are considered as prospective carriers for imaging agents and therapeutics due to their extended circulatory times, improved drug stability, specific targeting and proliferation into tumour tissue. Nanomicelles are employed as multifunctional molecular probes for identification (diagnosis), noninvasive screening and early treatment of ovarian cancer [72].

### 7.1. Diagnosis of Ovarian Cancer Employing Nanomicelles

Ovarian carcinoma is commonly identified in late stages due to comparative lack of early detection and diagnostic techniques in early stages [44]. The delivery and controlled release of therapeutics for site-specific targeted chemotherapy and imaging for early cancer identification are of great pertinence [76,77]. Imaging involves visualization of OC disease development, treatment efficacy and bio-distribution of therapeutics to the tumour, or investigation of molecular biomarkers [78]. Disease inspection and monitoring of therapeutic efficiency can be achieved by employing current medical visualizing modalities such as basic radiography, anatomical probes (CT scanning), ultrasound and magnetic resonance imaging (MRI) [74].

These imaging techniques can be categorized according to the energy utilized to develop visual images (heterogeneous X-ray beams, positron emissions, photon emissions), spatial specific resolution accomplished (macroscopic-, meso-scale, microscopic), or the nature of the captured information (anatomical, physiological or molecular/cellular imaging) [44,75]. However, these imaging techniques rely on a diagnosis of cancer when tumours have developed to approximately 1 cm^3^, and at this stage, the malignancy has around 1 billion metastatic tumour cells [79]. Furthermore, imaging probes have low signal transmission, instability, imprecise interactions, and rapid degradation from the circulatory system [80].

Nanotherapeutic applications incorporating noninvasive tumour molecular imaging have prospects in early prognosis by increasing the precision, efficacy of chemotherapeutics, and facilitating improved infection detection [44]. If image modalities are utilized to image tumours, improved tumour intensity is assimilated with contrast nanocarrier systems. Nanoparticles have distinct techniques for molecular-targeted delivery, drug encapsulation, or improvement of pathological areal imaging. Polymeric nanoparticles, including PEG-b-poly(Lysine) copolymers have great potential in analytic molecular imaging, monitoring of cancer development or regression [44]. Small particles within nanometer range, such as gold-plated-based molecules and coated metallic quantum molecules, are the most usually employed; however, other nanoparticles and biomarkers display possibilities as potent tools for potential transmission development and therapeutic delivery in diagnosis of infected sites [44]. Various one-off administered nanomicelle-based therapeutic delivery systems for tracking and targeting of ovarian cancer are outlined in Table 3.

Several nanomicellar technologies have been established and are presently undergoing extensive preclinical and clinical trials for application in chemotherapeutics and diagnostic imaging of ovarian cancer. Amphiphilic block-copolymers aggregate to form dual-layered nanomicelles and are future carriers of hydrophobic treatments and diagnostic probes. Partially soluble drugs and imaging agents are encapsulated into the inner core with hydrophilic surface of amphiphilic nanomicelles, forming a stable outer shell in an aqueous solution [77].

Diagnostic modalities for three main imaging probes are radioactive metals, including indium-111 (^111^ In), and radioactive metal complexes such as ortechnetium-99 m (^99 m^ Tc), used for scintigraphy; clustered/chelated magnetic metals, including gold, for magnetic resonance imaging (MRI); and iodine for conventional X-ray computed tomography (CT). The conventional contrast agents employed in medical therapeutics are low-molecular-weight complexes composed of these chemical probes. Several diagnostically significant amphiphilic composites have been effectively integrated into nanomicelles, including diethylene-triamine penta-acetic acid (DTPA), which are chelating agents for diagnostic imaging of various nanomicellar platforms utilized in MR diagnostic imaging. Polymeric nanomicelle systems, including iodine-containing PLL-PEG nanomicelles, are employed for cancer diagnostic imaging, utilizing conventional sectional tomography (CT) imaging and Single Photon Emission Computed Tomography SPECT using gamma rays. Furthermore, to monitor nanomicelles formulations and exchanges in cancer disease, nanomicelle co-encapsulated with imaging clustered/chelated metallic groups have been employed, for example, in gold compounds, manganese oxide-loaded nanoparticles, as well as being utilized with ultrasound (US) and magnetic resonance imaging (MRI) [76]. Currently, gadolinium (Gd)-contrast medium, including Magnevist^®^, are medically employed where visual contrast is increased by limiting the T1 reduction period (period of high longitudinal magnetization with brighter image) of aqueous protons. Integration of Gd compound on the nanomicelles’ surface upsurge the T1 reductivity and reactivity of diagnosis. The reactivity is improved by utilization of various developed architectural iron oxide nanoparticles such as surface designed Super Paramagnetic Iron Oxide Nanoparticles (SPIONS) that assemble in nanomicelle inner core and exhibit MRI reactivity at a nanomolar rate. Nanomicellar loading with therapeutics and imaging tools such as fluorescence Rhodamine and FITC probes are used for drug released imaging at specific tumour sites with distinctive designed image. Hence, nanomicelles are favourable as carriers for combinational chemotherapeutics and nanodiagnostic tools [98,99,100].

### 7.2. Treatment of Ovarian Cancer Using Nanomicelles

Nanomicelles are mainly administered intravenously (IV) and are usually exposed to several challenges of the blood circulatory system with resultant cytotoxicity before reaching the peritoneal cavity [101]. The intraperitoneal (IP) cavity is the principal site of OC disease [102]. Metastatic OC cells accountable for high mortality rate disseminate and recur at the intraperitoneal site [103]. Hence, IP nanomicellar chemotherapy is the favorable route of administration of OC treatment, with improved patient compliance as compared to intravenous (IV) treatment [104,105,106].

### 7.3. Targeting Strategies of Nanomicelles

**Figure 6:**
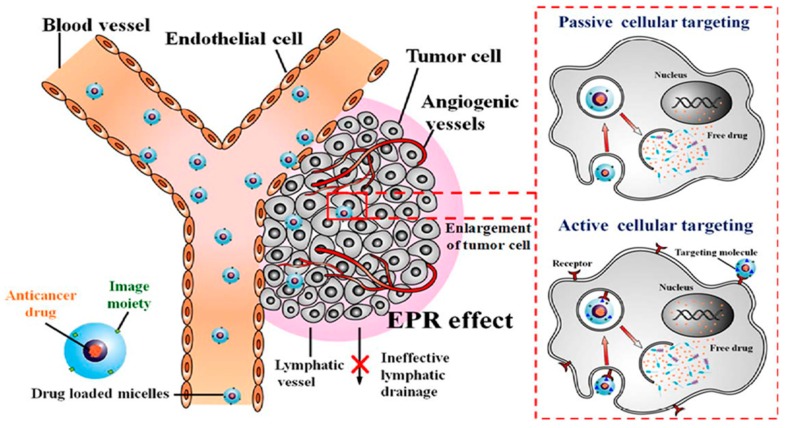
Schematic representation of drug loaded micelles (spheres) with imaging agents, from the administration site to the tumour tissue. After administration, micelles (10–200 nm) display specific targeting of tumour growth via passive targeting with cellular endocytotic uptake from exterior fluid to the cancer cells. Active targeting through receptor-mediated internalization is achieved by attachment of antibody ligand molecules, to the surface of micelles (Adapted with permission from Chen et al. [81]).

Targeted delivery of polymeric nanomicelles loaded with chemotherapeutic agents, present many diverse advantages [107]. Targeting is usually achieved using two delivery mechanisms as depicted in Figure 6; (i) passive targeting with improved vascular permeability and absorbency effect [39], (ii) specific active receptor-mediated targeting, employing ligand functionalized-nanomicelles, including the attachment of antibodies [63].

#### 7.3.1. Passive Targeting by Enhanced Permeability Effect of Tumour Tubular Blood Vessels

When nonfunctionalised nanomicelles have significant continual blood circulation period and successfully accumulate in tumour tissue through the passive enhanced permeability effect (EPR), this is indicative of passive targeting [76,108]. The therapeutic payloads are distributed to the tumour extracellular matrix and dispensed into the tumour cells and tissues. EPR targeting is ascribed to pathophysiological properties of tumours that are not identified in health tissue. These properties include the architecture of leaky tumour blood vasculature, impaired lymphatic drainage scheme, and increase in formation of permeability agents [109,110,111,112]. Several passive targeting nanocarrier systems have a PEG coating for stealth and “concealment” properties, including Genexol-PM, SP1049C, NK911, Opaxio™ (formerly Xyotax™), CRLX101, ProLindac™, SPI-77 and CPT-11 [76].

#### 7.3.2. Specific Active Receptor-Mediated Targeting

The active targeting approach involves the attachment of functional ligands to the nanomicelle surface. These ligands identify tumour-specific receptors that are overexpressed on the cancer cell plasma membranes, resulting in increased uptake and increased internalization of nanomicelles into tumour tissue via the receptor-mediated endocytosis process [113,114,115]. Commonly utilized affinity ligands are classified into the following categories: small unrefined molecules, nucleotides (RGD sequence), oligopeptides, sugar groups, folates, monoclonal antibodies (mAb), and nucleic DNA/RNA aptamers [116].

**Figure 7:**
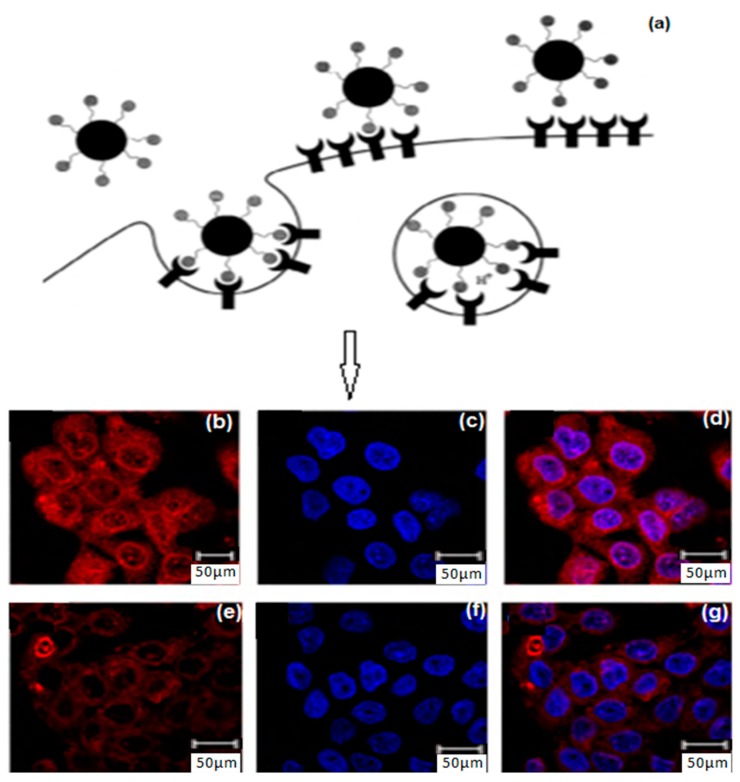
Schematic depiction of (**a**) active targeting, (**b**–**g**) confocal images of A431 cellular uptake incubated with cetuximab encapsulated micelles and lysotracker. The fluorescence intensity of A431 cells (**b**–**d**) treated with targeting micelles was 1.45 times higher than in cells incubated with antibody-free micelles (**e**–**g**) (Adapted with permission from Sudimack et al. [117]; Liao et al. [118].

There are several tools that are being utilized to target particles to tumour tissue. The use of an activating ligand is a dynamic approach that is reliant on specific receptors at an attachment site. These interactions include (glycoproteins/antibody), antigens and activating attachment groups (Figure 7). The “magic bullet concept” Ehrlich hypothesized that antibody-bounded nanocarriers have progressed into a model using three components: a therapeutic, a copolymer and active functionalizing agents associated with one formulation. This targeting therapeutic strategy provides rewards, including high target specificity for the pathological/infected area and minimal toxicity to the healthy cells. Furthermore, this therapeutic strategy also improves tumour treatment, chemotherapeutics of metastatic cancer of early stage carcinoma, when the primary papillary fallopian tubes are still immature [113,114,115].

## 8. Mucins as Targets for Antibodies in Chemotherapeutics

Most ovarian carcinomas are of epithelial origin and express mucins utilized as prospective diagnostics and treatment targets. Mucin glycoproteins are extracellular, glycosylated protein molecules, originating in the mucus coating and increased expression has been linked with various types of malignant pathology including OC. Currently, there are 20 identified mucins with two classifications: epithelial mucins (gelating, nongelating, film attached mucins) and lycoproteins (MUC9, MUC10, MUC18 and MUC20) [116,117,118]. Various research studies on the expression of mucin antigen in ovarian cancer have identified overexpression of film-attached mucins, especially MUC4, MUC5AC, and MUC16, but their biological applications are not defined. Mucin 16 (CA125) is used as a clinical biological marker in OC due to its elevated expression which results in CA125 release into the blood serum [119,120]. CA125 is a very huge cell surface mucin, first established by Robert Knapp in 1981. He identified this glycoprotein whilst exploiting identical monoclonal antibody-mAb [121]. Serum levels of CA125 are clinically utilized to diagnose OC patients on basis of regression or progression of the disease, subsequent to standard chemotherapy [122]. Moreover, abnormal mucin expression can trigger immunity and probably cause a strong antibody response. The antibody response is symptomatic of disease expression [123]. Immunoglobulin Ig (antibodies) affiliated with mucins, can have potential application in the progression towards the detection and therapy of ovarian cancer; however, there are still few studies conducted to date [121,122,123].

## 9. Stimulus-Responsive Nanomicelles

Stimuli-responsive nanomicelles (SRM) are smart nanoparticles engineered to respond to internal/ external stimuli of physical, chemical or biochemical origin, to control and release drug payloads at specific sites. SRM deliver drug payloads by structural alterations in response to the eliciting stimulus. The response presents with the degradation/disruption, polymerization or assembly of nanomicelles. The common internal stimuli in a cancer microenvironment are acidic pH, electrochemical redox potentials of the cell, and the availability of certain over-produced matrix enzymes, while external stimuli include temperature, attraction via magnetic field, light illumination (UV, visible, infrared) and ultrasound waves [95]. In this context, the formulation of nanomicelle, sensitive to external or internal stimuli is an alternative approach to targeted therapeutic release. In vitro models have provided evidence of progress for a number of stimuli-responsive approaches, however only a small proportion have been validated in animal preclinical prototypes, and also few (thermosensitive liposomes and iron oxide nanoparticles) are clinically approved by the FDA in terms of treatments and diagnostics [81].

## 10. Nanomicelles in Clinical Evaluations

Several therapeutic-loaded nanomicelles for chemotherapy have been evaluated for determination of toxicity and bioavailability [124]. While the impetus is on ovarian cancer, some examples of polymeric nanomicelles cited are for other types of cancer and applied for OC treatment. Preclinical evaluations and findings have revealed lots of positive data utilizing nanomicelles as therapeutic delivery systems for loading hydrophobic chemotherapeutic drugs [125]. Several micellar nanoformulations that are now under clinical evaluations are all stealth nanomicelle formulations, and specifically have a surface coating for stabilization to guarantee a compact conformational covering and protection against opsonisation by plasma proteins (Table 4) [69]. Genexol-PM micelle formulation is paclitaxel-loaded PEG-PLA micelle preparation [126]. NK012 micellar nanoformulation is also composed of a PEG coating with amino-acid repeat units, polyglutamate (PGlu) combined with antineoplastic 7-ethyl-10-hydroxy-camptothecin (SN-38) [69]. The hydrophobic PGlu component results in micelle aggregation. In vivo trials with NK012 micelle formulations validated the potency of antineoplastic action in a mice model. Recently, the accomplishments and applicability of NK012 were also screened in phase II trials in prominent breast tumour patients [69]. New paclitaxel (PTX) experimental formulations evaluated include NK105 that are composed of PEG coating and modified polyaspartate hydrophobic ration [69]. PTX drug is incorporated in the central core by hydrophobic links with the hydrophilic portion. Furthermore, a major decrease in cytotoxicity, from Cremophor EL and ethanol subsequent to primary PTX administration, was practical with NK105. In phase I trials with NK105 formulation, minor allergic reactions were identified in patients with bile duct, pancreatic, gastric, and colonic cancers compared to primary PTX treatment [69].

A SP1049C phase II cancer trial in cases with advanced stomach cancer has been conducted. SP1049C has been formulated as doxorubicin (DOX)-loaded Pluronic micelles [69]. In these phase II cancer trials, SP1049C displayed to be more effective than clinical doxorubicin in therapy of various types of carcinoma [69]. SP1049C displayed superior antineoplastic action, efficiency and increased in cancer cells in several pre-clinical carcinoma models as well as doxorubicin-resistant malignancies as compared to clinical doxorubicin [69]. SP1049C formulations have been screened in phase III trials in patients with metastatic adenocarcinoma of the gastrointestinal route [69]. To minimise toxicity and increase the efficacy of cisplatin, the micellar pharmaceutical preparation NC-6004 (Nanoplatin™) was developed. The NC-6004 consists of PEG coating with poly (γ-benzyl L-glutamate)/CDDP composite [69]. A small phase I pilot showed that NC-6004 was acknowledged by carcinoma patients that were affected by colorectal carcinoma, upper oesophageal carcinoma, and lung carcinoma [69]. The Genexol-PM formulation was a micellar PTX nanoformulation formulated from PEG with polylactic acid [68,134,135]. Preclinical in vivo trials with Genexol-PM formulation exhibited a threefold increase in average dissolution time and a significantly improved antineoplastic efficacy as compared with clinical paclitaxel [69].

## 11. Patents in Micellar Technologies for Targeted Chemotherapeutic Drug Delivery

In a patent by Kwon and associates (2015), solubilisation of cotton gossypol (a yellow, natural phenolic aldehyde plant pigment for inhibition of various dehydrogenase enzymes) with nanomicelles was conducted. Polymeric-nanomicelles integrated chemodrugs such as gossypol, and a combination of chemodrugs were evaluated, including mixture of a platinum-derived (cisplatin-(CDDP) or carboplatin) as well as a taxane (paclitaxel (PTX) or docetaxel-(DTX)), commonly used to cure nonsmall cell-lung (NSCLC) and ovarian cancers. The nanomicelle carrier’s composition enabled efficacious incorporation of the hydrophobic drugs [135,136,137]. Hence, this discovery provided stable and nontoxic biocompatible therapeutic formulations that potentially increased drug bioavailability. In another patent, nanomicelles encapsulating SN-38 formulation for chemotherapy of carcinoma were investigated. This development provided a nanomicelle formulation, including extended multiblock co-polymer with a SN-38 resulting from encapsulated camptothecin [61,138]. This SN-38 formulation is dominant over its camptothecin derivative since it is not reliant on stimulation by the detoxifying liver in vivo (Table 5) [138].

Bodrati in 2018 demonstrated the application of block-co-polymer nanomicelle of poly(oxyethylene)–block-poly(oxypropylene) copolymer in the administration of chemotherapeutic agents, providing noncovalent dissolvation, which minimized solubility issues [61]. Several copolymers are readily obtainable under the generic name of “poloxamers”/ “pluronics”. Innovation by Hao et al. (2017) comprised of nanomicelle aggregates, composites with self-aggregated/assembled nanomicelles and methods for formulating nanomicelle aggregates and composites. Nanoformulation also included a plant prolamin proteins attached to polyethylene glycol (PEG)-coated nanomicelle [139]. This invention further derived methods for integration of drugs utilizing the conjugates of the protein nanomicelle formulation. In a patent by Rhymer (2008) micellar structures, techniques of micellar assemblies, methods of nanoimaging, approaches of chemotherapeutic delivery and life biological composites were investigated [140]. This patent presented a therapeutic technique utilizing hydrophilic, high molecular mass block copolymer for facilitation of an intraperitoneally dosed antineoplastic agent for prolonged release in the peritoneal region. The patent further described a therapeutic-loaded nanomicelle formulation, consisting of a copolymer with an exterior water-soluble moiety, a polycarboxylic acid functional group; and an anti-tumour agent attached to or incorporated in the nanomicelle. Patents in micellar technologies for antineoplastic drugs delivery are presented in Table 5.

## 12. Future Recommendations

Nanomicelles are employed as drug carrier nanosystems or imaging agents. Extensive differentiation in physicochemical, pharmacological and immunological platforms is necessary prior to approval for application in humans. Antineoplastic efficacy of most chemotherapeutic nanoformulations has not advanced to an appropriate degree to evolve the formulated nanomedicines into clinical application. Thus, great research studies are conducted on optimization of physicochemical profiles of nanomicelles. Combinational chemotherapy against ovarian cancer is another approach used to enhance antineoplastic efficacy. Future trends in nanomicelle development and delivery includes circulatory computational evaluations, simulating ecosystems of the pathogens and patient-derived cell lines, induced pluripotent stem cell (iPSC) technology, three-dimensional coculture, organotypic systems, improvements in cell imaging, microfluidics, nanotechnologies and gene-editing technologies.

The main challenge is now linked with the interpretation of various productive and validated experimental findings into clinical translation. The efficacy of the therapeutics is limited due to degradation, interactions with cells, and poor tissue permeability. Furthermore, encapsulation of two or more therapeutics in a single nanocarrier system can be challenging due to different solubilities of the optimal drug combination. Nanotheranostics are therapeutics activated by a positive diagnosis of an ovarian cancer disease and will be in use in the near future for chemotherapeutics.

## 13. Conclusions

Novel nanomicellar technologies developed to date are focused on improving pharmacodynamics and pharmacokinetic profiles of the incorporated therapeutic agents, whilst increasing safety and compliance, to upsurge the five-year survival rate of OC patients. Nanomicellar systems have advanced as significant chemotherapeutic delivery platforms. These nanocarriers can be specifically loaded with a wide range of active drug compounds, providing a strategy to improve the bioavailability of drugs, including those abandoned due to insoluble characteristics and cytotoxicity challenges. Nanomicelles have also shown to be applicable for theranostic applications. Multipurpose polymeric nanomicelles have more attributes as therapeutic carriers, as shown by their considerable outcomes in the scope of clinical diagnosis and chemotherapeutics. These include nanomicelles attached with ligands, the enabling of specific active targeting of tumour metastasis, increased restorative effects, and reduced side effects—thus promoting more effective therapy. Although no panacea may be eminent at this time, it is anticipated that through tailored, safe, multifaceted, and rational design of nanomicelles, advanced drug delivery systems will be developed for the future treatment and diagnosis of OC.

## Figures and Tables

**Table 1:** Outline of the distinguishable nanotherapeutic tools designed for ovarian cancer treatment [19].

Nanosystems	Polymer–Drug Conjugates	Dendrimers	Polymer Micelles	Liposomes	Solid Lipid Nanoparticles
Size	≤10 nm	2–10 nm	10–100 nm	100–200 nm	50–1000 nm
Structural characteristics	Macromolecular structure	Macromolecular Tree-like structure	Spherical Supramolecular Core shell structure	Spherical bilayer vesicle structure	Spherical, bilayer-nanocapsular structure
Carrier composition	Water-soluble polymer	Hyperbranched polymer chains	Amphiphilic di and tri-block copolymers	Phospholipid, cholesterol membrane lipids	Solid lipid emulsifier water
Drug incorporation strategy	Covalent conjugation requiring functional groups on drug and polymer	Covalent conjugation requiring functional groups on drug and polymer	Noncovalent encapsulation/compatible with hydrophobic drugs	Noncovalent encapsulation/compatible with hydrophilic drugs	Noncovalent encapsulation/compatible with hydrophilic drugs
	PEG-paclitaxel & HPMA copolymer-doxorubicin—phase II trials SMANCS & CDP870 (Cimza)- Approved	Dendrimer- docetaxel & Viva gel- phase II & III trials PSMA-targeted dendrimers & Avidimer- dendrimers- Approved	CRLX- 101&NKTR-102- phase II/III clinical trials Genexol- PM- Approved	SGT53-01& MCC- 46 phase I clinical trials Doxil, Ambisome & DaunoXome- Approved	SLNs with [Gd-DTPA(H_2_O)]^2−^ and [Gd-DOTA(H_2_O)]^−^ compounds preclinical trials [31]. Diazemuls & Diprivan- Approved

**Table 2:** Building block copolymers employed in micelle drug transport nanosystems (adapted from Sutton et al., 2007) [69].

Copolymers	Abbreviation	Repeating Unit Structure
**Corona segment**		
Poly (ethylene glycol)	PEG, PEO	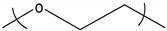
Poly (N-vinyl pyrrolidone)	PVP	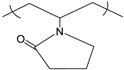
Poly (N-isopropyacrylamide	PNIPAM, NIPAM	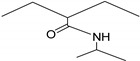
Poly (N-vinyl alcohol)	PVA	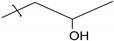
Poly (N-(2-hydroxyproyl methacrylamide)	pHPMAm	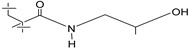
**Core segment**		
Polyesters		
Poly (propylene oxide)	PPO	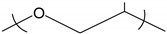
Poly esters		
Poly (L-lactide)		
Poly (D,L-lactide)	PLA, PDLLA	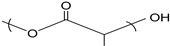
Poly (lactide-co-gycolide)	PLGA	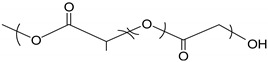
Poly (Ɛ-caprolactone)	PCL	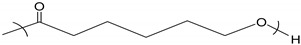
Poly(β-amino ester)		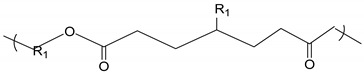
Poly(lactic acid)	PLA	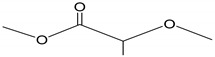

**Table 3:** Polymeric micellar systems employed for treatment and diagnosis (adapted from Kedar et al. [80]; Chen et al. [81]).

Polymer Structural Formula	Method of Synthesis	Method of Micellization	Delivered Agent	Mode of Delivery	References
PLGA-b-PPO-b-PLGA and PEG-b-PPO	Ring-opening polymerization	Dialysis method	Doxorubicin (DD)	P	[82]
Poly(ε-caprolactone)-b-PEO	Anionic ring opening polymerization	Dialysis method	Pyrene (hydrophobic fluorescent probe) (DA)	P	[83]
Poly(lactic acid)-polyurethane	Step condensation	Microphase separation method	Gliclazide (DD)	P	[84]
PMPC-b-PBMA	RAFT technology	Self-emulsion evaporation method	Paclitaxel (DD)	P	[85]
Poly(ethylene glycol-b-lactide)	Anionic ring opening polymerization	Oil-in-water emulsion method	Taxol (DD)	P	[86]
Poly(lactide-b-PEG)	Solvent polymerization	Self-emulsion solvent evaporation method	Paclitaxel (DD)	P	[87]
mPEG-b-p(HEMAm-Lac_n_)	Free-radical polymerization	Rapid heating procedure	Pyrene (DA)	P	[88]
ϒ-Benzyl l-glutamate N- Carboxyanhydride	Polymerization	Dialysis	Adriamycin (DD)	P	[89]
Acetal-PEG-b-PLA	Ring-opening polymerization	Dialysis method	Docetaxel, ^125^ I (DD), (DA)	Tyrosine-A, tyrosyl-glutmic acid-A	[90]
COOH-PEG-b-PLGA	Polymerization	Dialysis method	Docetaxel, paclitaxel (DD)	RNA aptamer-A DNA aptamer-A	[91]
PEG-b-PCL	Free-radical polymerization	Dialysis method	Paclitaxel, rapamycin (DD)	Folate- A	[92]
PEG-b-PLLA and P(HEMA)-b-p(His)	Solvent polymerization	Dialysis method	Doxorubicin (DD)	-	[93]
P(HEMA)-b-p(His)	Solvent polymerization	Dialysis method	Doxorubicin(DD)	Folate-A	[94]
PEG-b-PLA and HEMA-co-his)-g-PLA	Anionic ring opening polymerization	Oil-in-water emulsion method	Doxorubicin, Cy 5.5 (DD), (DA)	Folate-A	[95]
PEG-b-PLA and P(NVI-co-NVP)-g-PLA	Anionic ring opening polymerization	Oil-in-water emulsion method	Doxorubicin, ^123^ I (DD), DA	Folate-A	[96]
mPEG-b-PLA and P(NIPAAm-co-MAAc)-g-PLA	Solvent polymerization	Self-emulsion solvent evaporation method	Doxorubicin, FITC (DD), DA	Galactosamine-A	[97]

Abbreviations: P-passive targeting, A-active targeting, DD-Delivered Drug & DA-Delivered Agent.

**Table 4:** Polymeric micelle-based formulations containing chemotherapeutic drugs in clinical trials.

Formulation Trade Name	Incorporated Drug	Purpose	Polymer	Particle Size (nm)	Drug Loading (%)	Phase	References
Genexol-PM	Paclitaxel	Solubilization	MPEG-PDLLA	<50	16.7	III, IV	[127]
NK-105	Paclitaxel	Targeting	PEG-P(Asp)	85	23.0	II, III	[128]
SP-1049C	Doxorubicin	Anti-MDR effect	Pluronic L61, F127	30	8.2	I, II, III	[69]
DTXL-TNP	Doxorubicin	Targeting	PLA-PEG, PLA-PEG-ACUPA	100	10	I	[129]
NC-6004	Cisplatin	Targeting	PEG-P(Glu)-Cisplatin	30	39	I, II	[130]
NC-4016	DACH-platin	Targeting	PEG-P(Glu)-DACH-platin	20–100	25	I	[131]
NK 012	SN-38	Targeting	PEG-P(Glu)-SN38	20	20.0	II	[132]
NK911	Doxorubicin	Targeting	PEG-(Asp)-Dox	40	*n*. a	II	[133]

**Table 5:** Micellar patents issued in the area of cancer drug delivery (Adapted and modified from Mishra et al. [138]).

Patent Type	Title	Patent No.	Chemical Formula	Action	Year	Inventor/Assignee
Micelles	C6-c18-acylated derivative of hyaluronic acid	WO2014082609 A1	(HA)-[0(C^:^=O)NH-M]*_p_*	AC	2014	Contipro Biotech S.R.O.
Micelles	Polymer conjugated protein micelles	EP 2678001 A2	PEG-Prolamine	AC	2014	South Dakota State University
Paclitaxel Micelle (NK105)	Micellar preparation containing sparingly water-soluble anticancer agent and novel block copolymer	09705599.0	(poly(ethylene glycol)-copoly (L-aspartic acid)	AC	2013	Nanocarrier Co. Ltd. Nippon Kayaku Co., Ltd.
Nanoplatin^®^ (NC-6004)	Pharmaceutical composition and combined agent	098101554	(poly(ethylene glycol)-copoly (amino acid)	AC	2013	TOUDAI TLO Ltd.
DACH-Platin Micelle (NC-4016)	Coordination compound composed of diaminocyclohexane platinum (Ii) and block copolymer	2007-520209	(poly(ethylene glycol)-copoly (amino acid)	AC	2013	The University of Tokyo
Protein Micelle	Electrostatic bonding type macromolecular micelle drug carrier	EP2583563 A1	polyethylene glycol and poly(α,-β -aspartic acid)	AC	2013	TOUDAI TLO Ltd.
siRNA Micelle	Polyethylene glycol/polycation block copolymer	EP2087912 A1	PEG-PLys	AC	2013	The University of Tokyo
Sensor Linked Micelle	Active targeting polymer micelle encapsulating drug, and pharmaceutical composition	2008-539901	poly(ethylene glycol)-b-poly(2-aminoethyl methacrylate)-b-poly(styrene)	AC	2013	Nanocarrier Co. Ltd.
pH-Sensitive Micelle	Novel block copolymer used for preparing pH-responsive polymer micelles	2009-7007877	[PEG-p(Asp-Hyd-Adr)]	AC	2013	The University of Tokyo
Docetaxel Micelle	Docetaxel polymer derivative, method for producing same and use of same	2009250393	(mPEG-PDLLA)	AC	2013	Nanocarrier Co. Ltd.
Bortezomib Micelle	Pharmaceutical composition that includes block copolymer containing boronic acid compound	EP 2692777 A1	polyethylene glycol-polyglutamic acid	AC	2013	Nanocarrier Co. Ltd.
Micelles	Micelles for the solubilisation of gossypol	20120321715	Poloxamer or PEG-PCL	AC	2012	Wisconsin Alumni Research Foundation., US

Abbreviations: AC (Anticancer activity including ovarian cancer and various cancers such as lung and prostate cancer), MA Microaggregates), PEG/PEG 2000 (poly(ethylene glycol-2000), Hyaluronic acid (HA), C = O (carbonyl group), -PLys (polylysine), Asp(Aspartate), Hyd-Adr(hydrazone Adriamycin, poly-DL-lactide (PDLLA), PCL(polycaprolactone).
